# Accommodative Spasm Presenting as Pseudomyopia in a 47-Year-Old Male With Headache as the Primary Complaint: A Case Report

**DOI:** 10.7759/cureus.107533

**Published:** 2026-04-22

**Authors:** Rayan A Alzahrani, Mohammed O Elhorry, Asim Alghamdi, Saleh A Alghamdi

**Affiliations:** 1 Medicine, Faculty of Medicine, Al-Baha University, Al-Baha, SAU; 2 Ophthalmology, Dr. Saleh Abbas Specialized Polyclinic, Al-Baha, SAU; 3 Ophthalmology, Al-Baha University, Al-Baha, SAU

**Keywords:** accommodative spasm, cyclopentolate, cycloplegic refraction, headache, spasm of the near reflex

## Abstract

Pseudomyopia refers to an apparent myopic shift caused by accommodative overactivity that resolves after cycloplegia and may occur as an isolated accommodative spasm or within the broader spasm of the near-reflex spectrum. Because the refractive component is functional rather than structural, cycloplegic refraction is essential to confirm reversibility and avoid misclassification as true myopia.

A 47-year-old male police officer presented with a 1-month history of persistent, dull headaches associated with ocular discomfort. Symptoms were accompanied by intermittent nausea and visual strain, more pronounced during prolonged near tasks and smartphone use. Prior to an ophthalmologic assessment, he underwent a comprehensive neurological evaluation at a local hospital, including a computed tomography scan of the brain, which revealed no intracranial abnormalities. Cycloplegic refraction was performed in a staged manner across two visits due to occupational and driving requirements. At the initial visit, pre-cycloplegic autorefraction of the left eye demonstrated −0.75 −0.75 × 159° OS, with post-cycloplegic refraction of +1.25 −1.00 × 165° (+1.87 D hyperopic shift). At the two-week follow-up, pre-cycloplegic autorefraction of the right eye demonstrated plano −1.50 × 9° OD, with post-cycloplegic refraction of +1.75 −1.50 × 8° (+1.75 D hyperopic shift), suggesting a bilateral accommodative spasm. Near-addition spectacle correction was prescribed, and the patient was advised to limit prolonged near work, implement the 20-20-20 rule, and optimize workplace ergonomics.

Cycloplegic refraction remains central to the diagnosis of accommodative spasm and pseudomyopia, and a marked shift toward reduced myopia or hypermetropia after cycloplegia supports the diagnosis. In this patient, optical management with near-addition correction was preferred over pharmacologic cycloplegia because of occupational and driving requirements.

## Introduction

Pseudomyopia is a myopic refractive state in which an apparent myopic error resolves following cycloplegia [1]. This reversibility differentiates pseudomyopia from true myopia and highlights why cycloplegic refraction is fundamental in assessment. Multiple authors stress that cycloplegic refraction is needed to correctly identify pseudomyopia and avoid mislabeling it as structural myopia [2,3].

In the literature, pseudomyopia is commonly discussed within the broader spectrum of spasm of the near reflex (SNR), a condition characterized by excessive activation of accommodation, convergence, and pupillary miosis [3]. When accommodative spasm occurs without associated convergence spasm or miosis, it may present purely as pseudomyopia, whereas combined involvement of convergence and pupillary responses constitutes the full SNR triad [4]. Proposed mechanisms include parasympathetic overstimulation affecting accommodative control [4].

Reported manifestations include sudden blurred vision attributable to an apparent myopic shift, diplopia, variable ocular motility findings, ocular pain, photophobia, symptoms of eye strain, and headache [4,5]. Across studies, symptoms are described as episodic and fluctuating, and are often reduced or eliminated following cycloplegia [4].

Accommodative spasm is considered uncommon in clinical series of accommodative dysfunction. One report identified 4 cases among 114 pre-presbyopic patients, corresponding to approximately 2.5% [4]. Published cohorts largely involve children and young adults, with age ranges such as 7 to 33 years in one SNR series and 6 to 25 years in another accommodative spasm cohort [6,7]. Another review describes cases spanning a broader range from 8 to 41 years [3].

Here, we present a case of accommodative spasm presenting as headache in a 47-year-old male. Based on the available literature, published cohorts predominantly involve younger populations, and cases in patients approaching the fifth decade of life remain rarely reported. This case contributes to the growing recognition that accommodative spasm may occur in pre-presbyopic and early presbyopic adults, a group in whom the diagnosis may be overlooked or misattributed to the onset of presbyopia, potentially delaying cycloplegic evaluation and appropriate management.

## Case presentation

A 47-year-old male police officer presented to the ophthalmology clinic with a 1-month history of persistent, dull headaches associated with ocular discomfort. Symptoms were accompanied by intermittent nausea and visual strain that was more pronounced during prolonged near tasks and smartphone use. Prior to ophthalmologic assessment, the patient underwent a comprehensive neurological evaluation at a local hospital, including a computed tomography scan of the brain, which revealed no intracranial abnormalities. His medical history was unremarkable, with no known systemic illnesses or regular medication use. Ocular history was notable only for mild refractive error, with no prior ocular disease or surgery.

On examination, best-corrected visual acuity was 20/25 in the right eye (OD) and 20/30 in the left eye (OS). Intraocular pressure measured by Goldmann applanation tonometry (Haag-Streit AT 900; Haag-Streit Group, Köniz, Schweiz) was 14.0 mmHg OD and 19.7 mmHg OS. Slit-lamp examination revealed clear corneas, deep and quiet anterior chambers, round and reactive pupils without a relative afferent pupillary defect, and clear crystalline lenses bilaterally. Although gonioscopy was not performed, anterior chamber depth assessment using the Van Herick technique showed no features suggestive of angle closure. Fundus examination demonstrated healthy optic discs with cup-to-disc ratios of approximately 0.3, normal maculae, and unremarkable retinal vasculature and periphery.

Orthoptic evaluation showed normal ocular alignment at distance and near, with full extraocular motility in all gaze positions. There was no esodeviation at near, no limitation of abduction, and pupils were round and reactive with no documented miosis during examination, with no evidence of the full SNR triad on examination. Orthoptic findings are summarized in Table 1.

**Table 1:** Orthoptic assessment findings at Visit 1 RAPD: relative afferent pupillary defect

Orthoptic Parameter	Finding
Ocular alignment at distance	Orthophoric
Ocular alignment at near	Orthophoric, with no near esodeviation detected
Cover test (distance and near)	No manifest heterotropia
Extraocular motility	Full in all gaze positions bilaterally; no abduction deficit
Convergence spasm	Absent
Pupils	Equal, round, reactive OU; no RAPD; no miosis

Pre-cycloplegic autorefraction demonstrated myopic astigmatism: −0.75 −1.75 × 9° OD and −0.75 −0.75 × 159° OS. Given the patient’s symptom profile and extensive near-work demands, accommodative dysfunction with pseudomyopia was suspected. As the patient had driven a long distance to the clinic and required safe binocular vision for return travel, unilateral cycloplegic refraction using 1% cyclopentolate was performed in the left eye only after detailed counselling.

Post-cycloplegic refraction of the left eye, performed one hour after instillation, revealed a significant refractive shift to +1.25 −1.00 × 165°, representing a hyperopic shift of approximately +1.87 dioptres. This finding supported pseudomyopia due to accommodative spasm, with hyperopia unmasked under cycloplegia. Given the patient’s age, early presbyopia was considered alongside the accommodative spasm; a near-use spectacle correction was therefore prescribed at Visit 1 to reduce accommodative demand during prolonged near tasks. The patient was advised to limit prolonged near work, implement the 20-20-20 rule, and optimize workplace ergonomics. A follow-up was scheduled for two weeks later.

At the two-week follow-up (Visit 2), 14 days later, the patient reported mild improvement in headache severity but persistent ocular discomfort. He confirmed compliance with the prescribed near correction and lifestyle modifications. Intraocular pressure was 12.0 mmHg by applanation tonometry in both eyes, and anterior and posterior segment examinations remained unchanged. To complete the diagnostic evaluation, cycloplegic refraction was performed in the right eye only, per patient preference. Pre-cycloplegic refraction showed plano −1.50 × 9°, while post-cycloplegic refraction revealed +1.75 −1.50 × 8°, confirming a +1.75 dioptre hyperopic shift and bilateral accommodative spasm.

The spherical equivalent refraction (SER) of the non-cycloplegic right eye measurement improved from −1.625 D at Visit 1 to −0.75 D at Visit 2, a shift of +0.875 D toward the true cycloplegic value, following 2 weeks of near-addition wear and reduced near-work demands, without pharmacologic intervention. This spontaneous partial reduction in the apparent myopic shift is consistent with reduced accommodative overactivity after near-addition use and reduced near-work demand. A fixed structural refractive cause or uncomplicated latent hyperopia alone would be less likely to produce this pattern of inter-visit variability in the non-cycloplegic refraction. Cycloplegic refraction demonstrated a marked hyperopic shift in both eyes compared with pre-cycloplegic refraction (Table 2 ).

**Table 2:** Pre- and post-cycloplegic refraction findings Pre- and post-cycloplegic refraction findings and hyperopic shift in each eye. Cycloplegic refraction was performed unilaterally at each visit (OS at Visit 1, OD at Visit 2, 14 days later). Pre-cycloplegic values correspond to the visit at which each eye underwent cycloplegic evaluation. SER = spherical equivalent refraction = Sphere + (Cylinder ÷ 2). The hyperopic shift was calculated as the change in SER. For OD: pre-cycloplegic SER = −0.75 D, post-cycloplegic SER = +1.00 D, yielding a hyperopic shift of +1.75 D. For OS: pre-cycloplegic SER = −1.125 D, post-cycloplegic SER = +0.75 D, yielding a hyperopic shift of +1.87 D.

Eye	Visit	Pre-cycloplegic refraction	Post-cycloplegic refraction	Hyperopic shift (SER)
OS	Visit 1	−0.75 −0.75 × 159°	+1.25 −1.00 × 165°	+1.87 D
OD	Visit 2	plano −1.50 × 9°	+1.75 −1.50 × 8°	+1.75 D

The patient was advised to continue conservative management. At Visit 1, a near-use correction was prescribed to reduce accommodative demand during prolonged near tasks: +1.25 −1.00 × 11° OD and +1.25 −0.75 × 160° OS. At Visit 2, following completion of bilateral cycloplegic refraction, distance correction of +0.50 −0.75 × 5° OD and +0.50 −0.50 × 165° OS was prescribed, achieving best-corrected visual acuity of 20/20 in each eye. Relative to this distance correction, the near-use prescription issued at Visit 1 represented an effective addition of +0.75 D in each eye. Near visual acuity was 20/20 in each eye with and without the near correction; however, the patient reported greater comfort during near tasks with the addition in place, consistent with a reduction of the accommodative load as the underlying therapeutic mechanism. Spectacle prescriptions for both visits are summarised in Table 3. The patient declined further routine follow-up but was advised to seek reassessment if symptoms persisted or worsened.

**Table 3:** Spectacle prescriptions issued at Visit 1 and Visit 2

Visit	Prescription type	OD	OS	BCVA
Visit 1	Near-use only	+1.25 −1.00 × 11°	+1.25 −0.75 × 160°	—†
Visit 2	Distance	+0.50 −0.75 × 5°	+0.50 −0.50 × 165°	20/20 OU

At Visit 1, bilateral cycloplegic data were unavailable; a near-use prescription was issued without a distance component. BCVA with distance correction was formally assessed at Visit 2. Near visual acuity was 20/20 with and without the near addition at Visit 1.

## Discussion

The literature consistently places accommodative spasm within the spectrum of spasm of the near reflex, where accommodative spasm may occur alone or together with convergence spasm and pupillary miosis [3,4]. This framework helps account for the broad range of presentations, from isolated pseudomyopia to episodes accompanied by diplopia and ocular motility abnormalities [4].

The age at presentation in this case is atypical. Most published cohorts involve children and young adults, with reported age ranges of 6 to 25 years, 7 to 33 years [6,7], and 8 to 41 years [3]. Clinical suspicion for accommodative spasm is inherently lower in midlife patients, and the diagnosis may be misattributed to presbyopia onset. In this patient, headache was the dominant complaint, directing the initial workup along a neurological pathway that included unremarkable brain imaging. In this clinical context, the absence of structural intracranial or ocular pathology, together with near-task exacerbation, supported early consideration of cycloplegic refraction. At Visit 1, cycloplegic refraction was performed unilaterally in the left eye only, as the patient had driven a considerable distance and required safe binocular vision for return travel. This staged approach preserved functional vision while providing diagnostic confirmation; bilateral involvement was confirmed at Visit 2. Retinoscopy and subjective refraction were not performed; repeated autorefraction readings were stable and were used for documentation.

A key diagnostic feature is the discrepancy between non-cycloplegic and cycloplegic refraction: a more myopic non-cycloplegic result relative to the cycloplegic measurement indicates an accommodative rather than structural component [2]. In this case, the bilateral hyperopic shift (+1.87 D OS, +1.75 D OD) strongly supported pseudomyopia due to accommodative spasm, with hyperopia unmasked under cycloplegia. The main differentials considered were latent hyperopia alone, accommodative excess from sustained near work, and accommodative spasm within the SNR spectrum. While hypermetropes are known to demonstrate larger gap refractions than myopes [3], the accommodation reflex in uncorrected hyperopia functions to transiently relieve the manifest hypermetropic error, shifting the manifest refraction toward emmetropia, rather than producing apparent myopia [8,9]. It is specifically the state of accommodative spasm, arising from prolonged ciliary overactivity, that produces a shift toward myopia [8,9]. In this patient, the manifest refraction was myopic in both eyes despite the cycloplegic refraction being hyperopic in both eyes, indicating that accommodation did not merely compensate for the underlying hyperopic error but overshot emmetropia, producing apparent myopia [3,9]. This pattern is more consistent with accommodative spasm and is not adequately explained by uncomplicated latent hypermetropia alone. Accommodative excess, while plausible given the near-work history, predominantly affects younger patients, with excess ciliary tone typically amounting to approximately one dioptre [10]; by contrast, accommodative spasm produces prolonged ciliary contraction leading to varying degrees of pseudomyopia [10]. The cycloplegic shifts recorded in this patient (+1.75 D OD and +1.87 D OS) considerably exceed the approximately 1 dioptre of excess ciliary tone typically described in accommodative excess [10], consistent with a spasm rather than a simple excess mechanism. Furthermore, the non-cycloplegic spherical equivalent of the right eye improved spontaneously from −1.625 D at Visit 1 to −0.75 D at Visit 2 following two weeks of reduced accommodative demand, without pharmacologic intervention, demonstrating that the apparent myopia was dynamic and responsive to treatment, a pattern incompatible with a fixed structural or purely hyperopic refractive cause. The bilateral magnitude of the cycloplegic shifts, their reproducibility across two independent visits, and the spontaneous inter-visit improvement collectively support accommodative spasm as the diagnosis. The cycloplegic reversal argues against structural myopia [1,2].

Not all patients demonstrate the complete SNR triad. Some present as isolated accommodative spasm without esotropia or miosis [6]. As observed in this case, orthoptic assessment confirmed the absence of convergence spasm (Table 1 ) and no pupillary constriction. Partial SNR presentations of this kind are well-documented in the literature and do not preclude the diagnosis of accommodative spasm [6]. The objective cycloplegic shift provides the principal diagnostic support for a functional accommodative component. While functional or psychogenic causes are most commonly reported, infrequent associations with head trauma, neurological disease, and medications have been described [6].

Cycloplegia is central to both diagnosis and management. Pseudomyopia is defined by resolution of the apparent myopic error following cycloplegia [1,2], and in one clinical series, cycloplegic agents were employed for both diagnostic confirmation and treatment [7]. Management is individualized, as no standardized protocol exists [11]. Pharmacologic cycloplegia using atropine or cyclopentolate is described as an effective initial approach [5], while non-pharmacological measures, including plus lens correction and progressive addition lenses, are emphasized for longer-term control [5]. Near-addition spectacles were utilized as the primary management strategy for this patient. The use of a near-addition lens lowers the eye’s accommodative requirement below the actual demand of the near target, resulting in a proportional decrease in accommodative vergence [12]. This mechanism effectively reduces the ciliary contractile load and disrupts the sustained parasympathetic drive that perpetuates the spasm. This approach was preferred over pharmacologic cycloplegia given the patient’s occupational requirements as a police officer, which precluded the transient visual blurring and photosensitivity of cycloplegic agents during working hours. Pharmacologic cycloplegia was retained as an escalation option [5].

This case adds to a sparsely documented demographic in the accommodative spasm literature and illustrates the diagnostic risk in midlife patients not routinely offered cycloplegic refraction. The bilateral hyperopic shift was quantified and reproduced across two visits, lending diagnostic credibility despite the staged approach. Limitations include reliance on autorefraction without retinoscopy or documented subjective refraction, absence of gonioscopy, orthoptic assessment not repeated at follow-up, the non-use of modified optical fogging as an adjunctive measure, and a brief follow-up period that precludes assessment of long-term recurrence risk. While autorefraction is acknowledged to be less reliable than subjective refraction in accommodative disorders, the hyperopic shifts documented here (+1.75 D and +1.87 D) substantially exceed the variability typically attributable to the instrument alone. From a broader perspective, sustained near-work demands from digital device use are increasing across all age groups; a recent meta-analysis of 103 cross-sectional studies reported a pooled prevalence of computer vision syndrome of 69% among digital device users [13]. Further study is warranted to characterise the prevalence and outcomes of accommodative spasm in pre-presbyopic adults.

Overall, cycloplegic refraction remains the cornerstone for diagnosing accommodative spasm and pseudomyopia. A marked shift toward less myopia or hypermetropia following cycloplegia supports the diagnosis, and cyclopentolate or atropine can achieve adequate cycloplegia [7].

## Conclusions

This case highlights that accommodative spasm presenting as pseudomyopia can occur in midlife and may be missed when headache is the dominant complaint and neurological workup is pursued first. Prompt recognition and accurate assessment are key to avoiding treatment errors. A combined strategy incorporating both pharmacologic and non-pharmacologic measures is often used in clinical practice. Customized care plans, lifestyle modifications, and patient education are essential for preventing recurrence and protecting visual function in populations facing increasing digital visual demands.
